# A Systematic Review and Meta-Analysis Baduanjin Qigong for Health Benefits: Randomized Controlled Trials

**DOI:** 10.1155/2017/4548706

**Published:** 2017-03-07

**Authors:** Liye Zou, Jeffer Eidi SasaKi, Huiru Wang, Zhongjun Xiao, Qun Fang, Mark Zhang

**Affiliations:** ^1^Department of Sport Science, Hunan University, Hunan 410079, China; ^2^Department of Physical Education and Health Education, Springfield College, MA 01109, USA; ^3^Núcleo de Estudos em Atividade Física & Saúde (NEAFISA), Universidade Federal do Triângulo Mineiro, Avenida Tutunas, 490 Bairro Tutunas, 38061-500 Uberaba, MG, Brazil; ^4^Department of Sport and Physical Education, Shanghai Jiao Tong University, Shanghai 200240, China; ^5^Department of Foreign Language Teaching, Jishou University, Hunan 416000, China; ^6^Department of Sport Management, Delaware State University, Dover, DE 19901, USA

## Abstract

*Objective*. To investigate the effects of practicing Baduanjin Qigong on different health outcomes.* Methods*. Six electronic databases were used for literature search through entering the following key words: Baduanjin Qigong, quality of life, sleep quality, and health-related outcomes.* Results*. Nineteen randomized controlled trials were used for meta-analysis. The aggregated results from this systematic review have shown significant benefits in favour of Baduanjin Qigong on quality of life (SMD, −0.75; 95% CI −1.26 to −0.24; *P* = 0.004), sleep quality (SMD, −0.55; 95% CI −0.97 to −0.12; *P* = 0.01), balance (SMD, −0.94; 95% CI −1.59 to 0.30; *P* = 0.004), handgrip strength (SMD, −0.69; 95% CI −1.2 to −0.19; *P* = 0.007), trunk flexibility (SMD, −0.66; 95% CI −1.13 to −0.19; *P* = 0.006), systolic (SMD, −0.60; 95% CI −0.94 to −0.27; *P* = 0.0004) and diastolic blood pressure (SMD, −0.46; 95% CI −0.73 to −0.20; *P* = 0.0005), and resting heart rate (SMD, −0.87; 95% CI −1.47 to −0.27; *P* = 0.005). The aggregated results of meta-analyses examining the effect of Baduanjin Qigong on leg power, cardiopulmonary endurance, and pulmonary function remain unclear because of a small number of studies.* Conclusions*. The aggregated results from this systematic review show that Baduanjin Qigong practice is beneficial for quality of life, sleep quality, balance, handgrip strength, trunk flexibility, systolic and diastolic blood pressure, and resting heart rate. Further studies are necessary to confirm the effects of Baduanjin Qigong on leg power, cardiopulmonary endurance, and pulmonary function (e.g., vital capacity), while considering a long-term follow-up.* Registration Number*. This trial is registered with International Prospective Register of Systematic Reviews (PROSPERO): CRD42016036966.

## 1. Introduction

Baduanjin (also called Eight-Section Brocades) is one of the forms of traditional Chinese Qigong exercises which has a history of more than 1000 years. It is characterized by interplay between symmetrical physical postures and movements, mind, and breathing exercise in a harmonious manner. Baduanjin Qigong is comparatively easy to learn with less physical and cognitive demands because it only contains eight simple movements created based on the traditional Chinese medicine theory [1, 2]. Its primary focus is on the release of internal body energy with the intent of producing diverse health benefits [3, 4]. Internal techniques of Chinese martial arts, including different forms of Qigong (e.g., Tai Chi Chuan, Baduanjin, and five mimic-animal exercises), have gained more and more popularity worldwide since the Chinese Health Qigong Association was established in 2004 to strive for promotion of Qigong [5, 6].

As the number of Baduanjin Qigong practitioners has grown in different parts of the globe, it has the number of scientific studies on the health benefits resulting from practicing Baduanjin Qigong. These studies have examined the effects of Baduanjin Qigong on different aspects of health, including mental health [7–9], cardiovascular parameters [10, 11], quality of life [9–11], sleep quality [11–14], osteoarthritis [2, 15], cardiorespiratory fitness [16, 17], physical performance [9, 11], balance [18], and flexibility [19]. While evidence arising from these studies indicates that Baduanjin Qigong is beneficial for health, only a few systematic studies have been conducted to summarize the results from these studies. In addition, the systematic reviews were conducted for specific health conditions/parameters, including hypertension [12], type 2 diabetes [20], pain [21], and blood lipid metabolism [22].

Considering the substantial number of studies produced over the last decade on the health benefits of practicing Baduanjin Qigong, it is valuable for the research community to have access to a comprehensive review and summary of study results. The previous reviews on the topics were mainly focused on the effectiveness of Baduanjin Qigong on physiological responses (e.g., blood glucose, triglyceride, low-density lipoprotein-cholesterol, and blood lipid metabolism) [12, 22]. However, a comprehensive review has not been conducted to specifically evaluate the effectiveness of Baduanjin Qigong on other health-related outcomes (e.g., sleep quality, quality of life, and physical fitness) in both healthy and special populations. In addition, meta-analysis as the highest level of evidence on the evidence hierarchy is more convincible because researchers can utilize randomized controlled trials to develop a more correct estimate of Baduanjin Qigong effect magnitude [23]. Therefore, we conducted a systematic review and meta-analysis of studies, to determine whether practicing Baduanjin Qigong is beneficial for different health outcomes.

## 2. Methods

### 2.1. Search Strategy

Six electronic databases (PubMed, Cochrane Library, WangFang, Google Scholar, Chinese National Knowledge Information Database [CNKI], and Physiotherapy Evidence Database [PEDro]) were used for literature search, along with the following search terms in different combinations: Baduanjin Qigong, quality of life, sleep quality, blood pressure (systolic and diastolic), heart rate, and components of physical health (e.g., physical balance, flexibility, and handgrip strength). A follow-up from reference lists of relevant articles identified was also performed. A review author (HRW) was responsible for contacting with Chinese Qigong experts in order to gain a comprehensive understanding of Baduanjin Qigong. The bibliographies of included studies for additional references were manually identified. The Preferred Repointing Items for Systematic Reviews and Meta-Analyses (PRISMA) was used to present detailed information in this systematic review and meta-analysis [24].

### 2.2. Eligibility Criteria

The studies were included if they met the following criteria: (1) randomized controlled trials; (2) peer-reviewed studies in English or Chinese; (3) original studies published from 2002 to 2016; (4) Baduanjin Qigong as the main intervention in the studies; (5) a minimum of one outcome measure relating to quality of life, sleep quality, blood pressure, heart rate, and components of the physical health [the review authors extracted the most commonly reported outcomes]; (6) participants who must be adults. The studies were excluded if (1) a combination of Baduanjin Qigong and other exercises as a primary intervention was used; (2) with respect to the quality of life and sleep quality, the sum scores were not reported; (3) full-text articles did not report the detailed information needed for meta-analysis (e.g., mean and standard deviation at baseline and postintervention or within-group change score from baseline); (4) sample size in the study was smaller than 20.

### 2.3. Methodological Quality Assessment

The two reviewers (LYZ and FQ) carried out methodological quality assessment of eligible studies using a standard PEDro scale. The PEDro scale is comprised of the following elements: eligibility criteria, random allocation, concealed allocation, being similar at baseline, subjects blinded, therapists blinded, being smaller than 15% dropout, intention-to-treat-analysis, between-group comparison, point measure and variability, and assessors blinded (if self-reported outcomes [e.g., quality of life and sleep quality] in those studies were measured, the assessor is considered to be blind). If a criterion was satisfied, a point (one) is awarded for the study and vice versa (zero). For each study included, a PEDro sum score ranging from 1 to 10 could be obtained without taking into account the eligibility criteria, with higher scores indicating better methodological quality. The sum score is classified methodological quality of each study into (1) poor quality = PEDro score ≤ 3; (2) fair quality = PEDro score between 4 and 5; (3) good quality = PEDro score between 6 and 10 [25–27].

### 2.4. Data Extraction

Two reviewers (LYZ and FQ) independently extracted data based on a predetermined data extraction form consisting of basic information (name of author, year of publication, study design, sample size, mean and standard deviation for age, number of participants in study groups, outcomes measured, adverse events, and follow-up assessment) and detailed information relating to the interventions (frequency and duration and comparison details). A third party appeared and had dealt with disagreement between the two reviewers.

With respect to the within-group change scores from baseline, if authors did not report the change score data, the reviewers (xxx) first tried to contact the authors via email or phone call to obtain the data. In cases where the data was not obtainable, reviewers used one of the following methods: (1) if no significant difference on the outcomes at baseline between two groups existed, postintervention scores were used for data analysis; (2) if baseline scores were significantly different, reviewers attempted to estimate the change scores and standard deviation through standard formulas provided by Cochrane Handbook for Systematic Reviews of Interventions [28]. If reviewers were unable to find the relevant information for estimating the change scores, the study was excluded.

### 2.5. Statistical Analysis

Revman 5.3 software within the Cochrane Collaboration for data analysis was used to synthesize the outcomes (e.g., quality of life, sleep quality, blood pressure, heart rate, and components of the physical health) from the randomized controlled trials. Due to quality of life and sleep quality measured by a variety of scales in the studies included, standardized mean difference (SMD) is more appropriate to be calculated in the meta-analysis, along with a more conservative random-effects model for testing heterogeneity, while 95% confidence intervals (CI) were set [29–31]. A value of *I*^2^ statistics with 50% as a cutoff point was used to evaluate consistency of the outcome measures across the studies included. If the value of *I*^2^ statistics was greater than 50%, it indicates an existence of the heterogeneity. In this way, sensitivity analysis was used through removing the inappropriate study. In addition, by study with one Baduanjin Qigong intervention and two comparison groups (non-Baduanjin Qigong intervention and control groups), reviewers kept the control group but removed the non-Baduanjin Qigong group.

The method is recommended by Cochrane Handbook for Systematic Reviews of Interventions 16.5.4 (how to include multiple groups from one study) [28].

## 3. Results

### 3.1. Literature Search

A total of 274 relevant records were identified through the six search databases.

**Figure 1:**
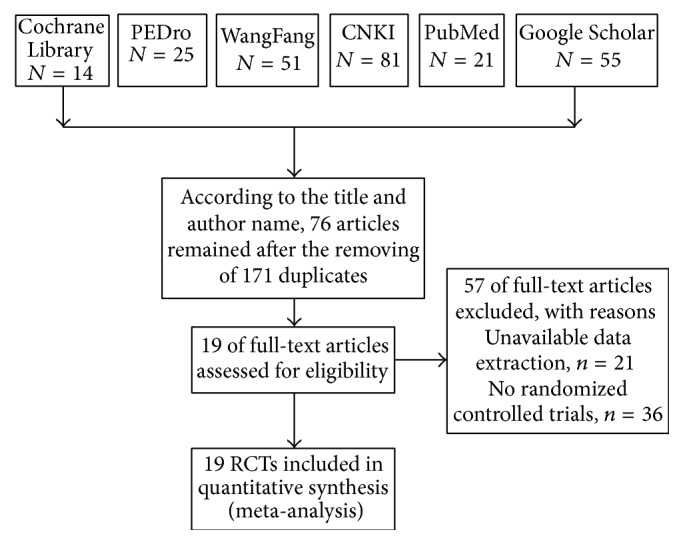
Flowchart showing the retrieval of studies for review. PEDro = Physiotherapy Evidence Database; CNKI = Chinese National Knowledge Information Database; RCT = randomized controlled trial.

According to the title and author name, 76 articles remained after removing the 171 duplicates. And 57 full-text articles were excluded because of the reasons, including unavailable data extraction (e.g., sum score relating to quality of life or sleep quality was not obtainable) (*n* = 21) and no randomized controlled trials (*n* = 36). The final number of 19 randomized controlled trials (RCT) was used for meta-analysis. Of these, five studies were published in English and fourteen in Chinese. The flowchart showing the retrieval of studies for this review is presented in the Figure 1.

### 3.2. Study Characteristics

The characteristics of the 19 RCTs included are presented in Table 1. These studies were published between 2008 and 2015 (please see the first column of Table 1 presenting the year of each publication). A total of 1535 participants (an age range from 19 to 75 years) were included in this review, including 559 adults with healthy status and 976 with different types of diseases (e.g., type 2 diabetes mellitus, cancer, Parkinson's disease, hypertension, knee osteoarthritis, and chronic fatigue syndrome-like illness). Sample size in the eligible studies ranged from 20 to 222. When compared to Baduanjin Qigong intervention groups, study participants in the control groups were asked to keep either their original lifestyle [9, 13, 17, 18, 20, 32–35], educational lessons relating to diseases [14, 15, 36, 37], regular drug treatment [38, 39], regular healthcare [2, 40], or daily self-walking [11, 41]. Study participants in the Baduanjin Qigong intervention groups (intervention period ranging from 4 to 24 weeks) experienced Baduanjin Qigong exercise duration ranging from 30 to 90 minutes, along with the frequency of weekly sessions from 2 to 7. After Baduanjin Qigong intervention period, follow-up assessment was only performed by two studies (reporting no adverse events), involving three [13] and twelve weeks [9], respectively.

### 3.3. Methodological Quality

The PEDro scores of 19 RCTs are presented in Table 2. Removing score from eligibility criteria in each study, the sum PEDro score ranged from 4 to 7 points (fair-to-good methodological quality). Concealed allocation, blinding of participants, and blinding of therapists were not observed in all RCTs, which is acceptable because of nonpharmacological clinical trials [42–44]. Blinding of assessors was present in 12 RCTs [9, 11, 13–15, 20, 32, 35–37, 40, 41] but not employed in 7 RCTs [2, 17, 18, 33, 34, 38, 39]. Of 19 RCTs, only four RCTs were observed that the dropout rate was greater than 15% [2, 20, 32, 36]. Intention-to-treat analysis was not employed in seven RCTs because the authors did not consider the participants who withdrew for data analysis [2, 11, 17, 19, 32, 36, 38]. The remaining criteria of the PEDro scale in all RCTs were reported to have high methodological quality.

### 3.4. Meta-Analysis of Outcomes Measured

**Figure 2:**
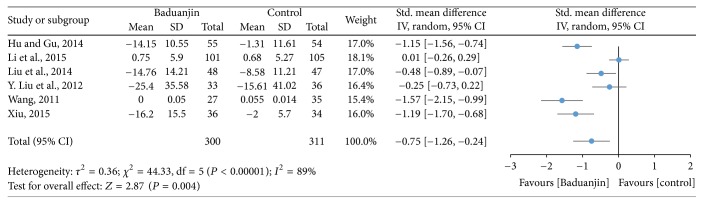
Forest plot showing the effect of Baduanjin Qigong on quality of life.

For the meta-analysis, six studies (a total of 611 participants) identified the effect of Baduanjin Qigong on quality of life measured using assessment tools with high reliability and validity, including SF-36 [11, 18], CSWQ [7], DMQIS [36], WHOQOL [9], and EORTC-QLQ-C30 [40]. A higher negative value of mean score for the tests indicates better quality of life, whereas a higher positive value of mean score for the tests indicates the worse quality of life. The aggregated result has shown a significant benefit in favour of Baduanjin Qigong on quality of life (SMD, −0.75; 95% CI −1.26 to −0.24; *P* = 0.004; Figure 2).

**Figure 3:**
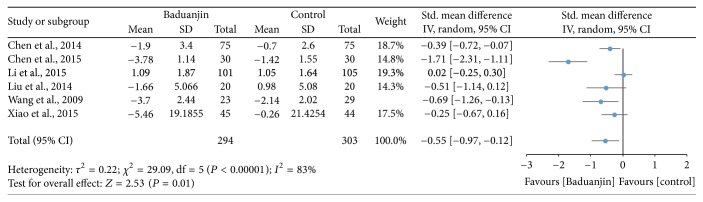
Forest plot showing the effect of Baduanjin Qigong on sleep quality.

Six studies (a total of 597 participants) identified the effectiveness of Baduanjin Qigong on sleep quality measured using assessment tools with high reliability and validity, including PSQI [9, 13–15, 32] and PDSS-2 [41]. A higher negative value of mean score for the tests indicates better sleep quality, whereas a higher positive value of mean score for the tests indicates the worse sleep quality. The overall result of the meta-analysis showed that Baduanjin Qigong is associated with significantly improved sleep quality (SMD, −0.55; 95% CI −0.97 to −0.12; *P* = 0.01; Figure 3).

**Figure 4:**
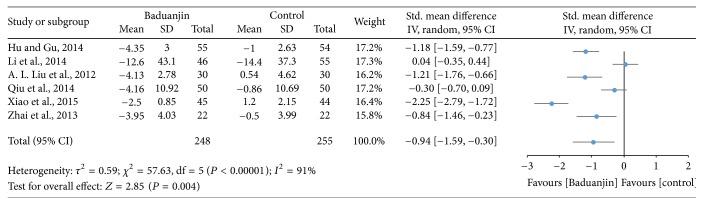
Forest plot showing the effect of Baduanjin Qigong on physical balance.

Six studies (a total of 503 participants) examined the effect of Baduanjin Qigong on physical balance measured using stork balance test [17, 18, 33–35] and Berg Balance Scale [41]. A higher negative value of mean score for the tests indicates better physical balance, whereas a higher positive value of mean score for the tests indicates the worse physical balance. The overall result of the meta-analysis showed that Baduanjin Qigong is associated with a statistical improving on physical balance (SMD, −0.94; 95% CI −1.59 to −0.30; *P* = 0.004; Figure 4).

**Figure 5:**
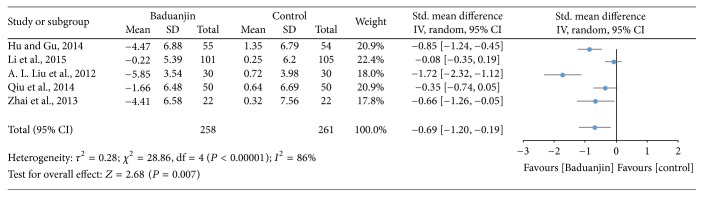
Forest plot showing the effect of Baduanjin Qigong on handgrip strength.

Five studies (a total of 519 participants) investigated the effect of Baduanjin Qigong on handgrip force measured using standard handgrip dynamometer [9, 18, 33–35]. A higher negative value of mean score for the tests indicates stronger handgrip strength, whereas a higher positive value of mean score for the tests indicates the worse handgrip strength. The aggregated result has shown a significant benefit in favour of Baduanjin Qigong on handgrip strength (SMD, −0.69; 95% CI −1.2 to −0.19; *P* = 0.007; Figure 5).

**Figure 6:**
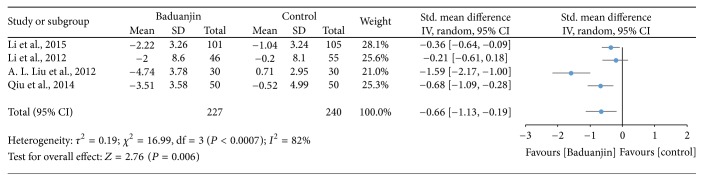
Forest plot showing the effect of Baduanjin Qigong on trunk and hip flexibility using sit-and-reach test.

Four studies (a total of 467 participants) examined the effect of Baduanjin Qigong on trunk and hip flexibility measured using sit-and-reach test [9, 20, 33, 35]. A higher negative value of mean score for the tests indicates better trunk and hip flexibility, whereas a higher positive value of mean score for the tests indicates the worse trunk and hip flexibility. The aggregated result of the meta-analysis has shown a significant improvement in favour of Baduanjin Qigong on trunk and hip flexibility (SMD, −0.66; 95% CI −1.13 to −0.19; *P* = 0.006; Figure 6).

**Figure 7:**
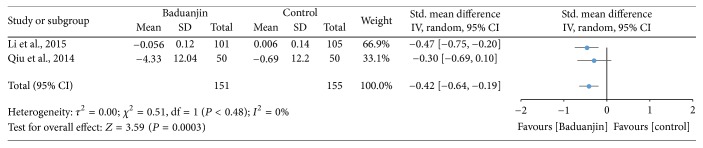
Forest plot showing the effect of Baduanjin Qigong on leg power measured using standing long jump test.

Two studies (a total of 306 participants) examined the effect of Baduanjin Qigong on leg power measured using standing long jump, with long distance indicating strong leg power [9, 34]. A higher negative value of mean score for the tests indicates better leg power, whereas a higher positive value of mean score for the tests indicates the worse leg power. The aggregated result of the meta-analysis has shown a significant improvement in favour of Baduanjin Qigong on lower extremity power (SMD, −0.42; 95% CI −0.64 to −0.19; *P* = 0.0003; Figure 7).

**Figure 8:**
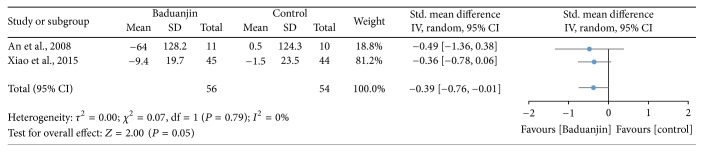
Forest plot showing the effect of Baduanjin Qigong on six-minute walking test.

Two studies (a total of 110 participants) examined the effect of Baduanjin Qigong on six-minute walking performance [2, 41], with long distance indicating better aerobic endurance. A higher negative value of mean score indicates better aerobic endurance, whereas a higher positive value of mean score for the tests indicates the worse aerobic endurance. The aggregated result of the meta-analysis has shown a significant benefit in favour of Baduanjin Qigong on six-minute walking performance (SMD, −0.39; 95% CI −0.76 to −0.01; *P* = 0.05; Figure 8).

**Figure 9:**
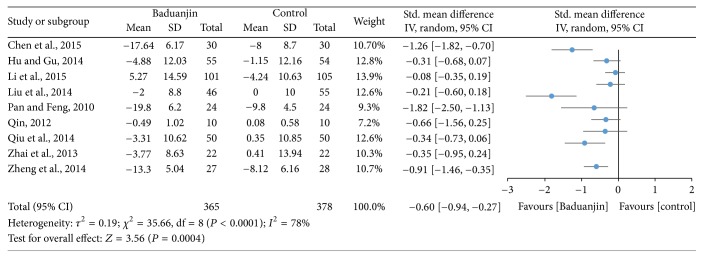
Forest plot showing the effect of Baduanjin Qigong on systolic blood pressure.

**Figure 10:**
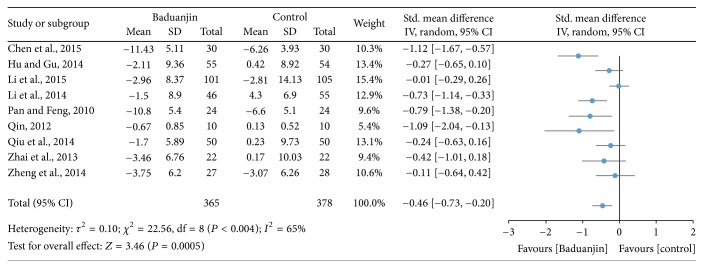
Forest plot showing the effect of Baduanjin Qigong on diastolic blood pressure.

Nine studies (a total of 743 study participants) examined the effect of Baduanjin Qigong on blood pressures which were all measured at quiet condition [9, 17, 18, 20, 33, 34, 37–39]. Systolic and diastolic blood pressures were reported in the nine identical studies. A higher negative value of mean score indicates better SBP and DBP, whereas a higher positive value of mean score for the tests indicates the worse SBP and DBP. The SBP (SMD, −0.60; 95% CI −0.94 to −0.27; *P* = 0.0004) and DBP (SMD, −0.46; 95% CI −0.73 to −0.20; *P* = 0.0005) were reported, respectively, in Figures 9 and 10, which has shown significant decrease at quiet condition in favour of Baduanjin Qigong on both the SBP and DBP.

**Figure 11:**
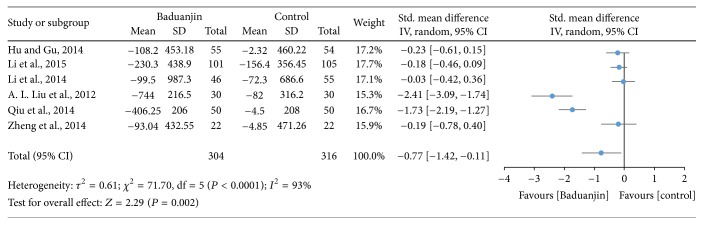
Forest plot showing the effect of Baduanjin Qigong on respiratory efficiency measured using vital capacity.

Six studies (a total of 620 participants) investigated the effect of Baduanjin Qigong on respiratory efficiency using vital capacity at quiet condition [9, 18, 20, 33–35]. A higher negative value of mean score indicates better respiratory efficiency, whereas a higher positive value of mean score for the tests indicates the worse respiratory efficiency. The results of meta-analysis showed a significant improvement in favour of Baduanjin Qigong on vital capability (SMD, −0.77; 95% CI −1.42 to −0.11; *P* = 0.02; Figure 11).

**Figure 12:**
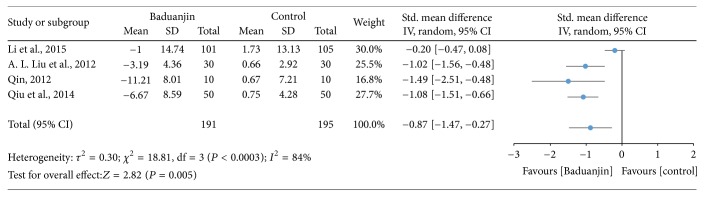
Forest plot showing the effect of Baduanjin Qigong on cardiorespiratory endurance measured using resting heart rate.

Four studies examined the effect of Baduanjin Qigong on cardiorespiratory endurance measured using resting heart rate [9, 17, 34, 35]. A higher negative value of mean score indicates better cardiorespiratory endurance, whereas a higher positive value of mean score for the tests indicates the worse cardiorespiratory endurance. Comparing to the control groups, the results of meta-analysis showed a significant improvement in favour of Baduanjin Qigong on cardiorespiratory endurance by reducing resting heart rate (SMD, −0.87; 95% CI −1.47 to −0.27; *P* = 0.005; Figure 12).

## 4. Discussion

### 4.1. Quality of Life

Of the six studies reviewed, two [9, 36] did not show significant effects of Baduanjin Qigong on quality of life, while the other four did [7, 11, 17, 40]. The aggregated results from our meta-analysis showed that Baduanjin Qigong is beneficial for quality of life. The magnitude of effects was statistically significant, indicating that Baduanjin Qigong is an exercise modality that may be used as a strategy for promoting quality of life. The benefits of Baduanjin Qigong on quality of life are more evident on older adults and individuals with chronic conditions, possibly because these populations usually demonstrate a lower functional capacity, being more likely to benefit from light intensity exercise. Positive results were observed with training regimens of at least two 30-minute weekly sessions [17]. According to the studies included in the meta-analysis, the minimum duration of the interventions for beneficial results on quality of life was 8 weeks [7, 40]. It is important to highlight that, of the studies included in the analyses, none used interventions lasting for less than 8 weeks. Therefore, we cannot rule out the possibility that shorter Baduanjin interventions may also result in improved quality of life. It is also important to mention that the studies included in this review used different instruments to assess quality of life and we only examined the total scores of these instruments. It is possible that Baduanjin Qigong may influence the various facets of the quality of life construct differently.

### 4.2. Sleep Quality

In this systematic review we found that the pooled results from randomized controlled trials indicate that Baduanjin Qigong is effective in improving sleep quality. While statistically significant, the pooled standardized mean difference was greater than 0.5, suggesting that overall the effect of Baduanjin Qigong on sleep quality is moderate. Of the three studies presenting significant results of Baduanjin Qigong on sleep quality [13, 14, 32], one included educational lessons for reducing hypertension [14]. This study was the one presenting the greatest effect size. If the educational lessons were not included in the study, it is possible that the magnitude of the effect size could be smaller, which could also result in a smaller pooled standardized mean difference between Baduanjin Qigong and the control condition. On the other hand, the results from Chen et al. [14] suggest that, in order to maximize positive results of Baduanjin Qigong on sleep quality, health professionals may use the strategy of combining educational lessons with training sessions. Due to the small number of studies from this review demonstrating positive results for sleep quality, we cannot speculate on optimal parameters for training duration and frequency and intervention duration. Furthermore, the studies included in this systematic review only looked at the effects of Baduanjin Qigong practice on sleep quality in patients with chronic conditions. We cannot ascertain if Baduanjin Qigong is effective in improving sleep quality in healthy individuals. However, other studies in the literature, many studies, have shown beneficial effects of physical activity on sleep quality [45–47], including studies with Tai Chi Chuan [48–50], a modality with similar characteristics of the Baduanjin Qigong. Future studies may help to better elucidate the magnitude of the effects of Baduanjin Qigong on sleep quality. For the present review, we only found six studies that met the inclusion criteria. Finding exercise modalities that may alleviate stressful routines of modern life is important to reduce impacts on sleep quality.

### 4.3. Physical Fitness

Of the six studies included in this systematic review, five demonstrated positive effects of the Baduanjin Qigong practice on physical balance [17, 18, 33–35, 41], with three of these studies showing standardized mean differences greater than 1 [17, 18, 35, 41]. The meta-analysis of the six studies showed significant beneficial effects of the Baduanjin Qigong on physical balance. These beneficial effects were observed for young [18, 33] and older adults [17, 35, 41], as well as for individuals with Parkinson's disease [41], with more pronounced benefits in the latter group. Improving balance is of major importance for preventing falls in older adults and individuals with neurodegenerative diseases, such as Alzheimer's disease and multiple sclerosis [51–55]. In this context, many studies have used Tai Chi Chuan as an intervention strategy with these populations. These studies have consistently found positive effects of Tai Chi Chuan on balance, emphasizing it as an appropriate modality for individuals with balance impairment [56–59]. The results of the present systematic review suggest that Baduanjin may be an equally effective alternative for individuals desiring to improve balance. According to the results from this meta-analysis, beneficial effects of Baduanjin Qigong on physical balance can be observed with training programs including at least four sessions/week of 30–60 minutes of practice [17]. Eight weeks of intervention is already effective in promoting improvements in balance [33].

Muscular strength is another component of physical fitness that has been investigated in studies with Baduanjin Qigong. In this meta-analysis, muscular strength results were pooled for two commonly used tests: handgrip strength test and standing long jump test. For the former, three of the five studies included in this review demonstrated significant results of Baduanjin Qigong practice on handgrip strength [18, 33, 35]. The greatest standardized mean difference was observed for the study of [35] with a value close to 2 (SMD: −1.72; 95% CI: −2.32, −1.12).

The pooled standardized mean difference of the five studies was −0.69 (95% CI: −1.20, −0.90), denoting a statistically significant medium effect of Baduanjin Qigong practice on handgrip strength. Based on the results, it is possible to infer that Baduanjin Qigong is effective in improving handgrip strength. Improvements in handgrip strength were observed for young and older adults, with greater magnitude for the former group [35]. It appears that a training regimen of as low as four sessions/week of 30–60 minutes is already effective for increasing handgrip strength [17]. Benefits may be observable with eight weeks of Baduanjin Qigong practice [33]. A possible explanation for the improvements in handgrip strength is the performance of upper body isometric exercises during regular Baduanjin Qigong routines. In addition, there may be potential improvements in muscle recruitment patterns as the training regimen progresses.

Ortega et al. [60] suggested that physical fitness should be taken into account as a powerful marker of health. Therefore, leg power is one of essential components of the physical fitness that can be used as a health status indicator. In relation to the effects of Baduanjin Qigong on the performance of the long jump test, we only found two studies meeting the inclusion criteria. Although the pooled standardized mean difference for the effects of Baduanjin Qigong on the score of the long jump test was significant (SMD: −0.42; 95% CI: 0.64, −0.19), it is still difficult to draw a confirmative decision based on a small number of studies [9]. Thus, more studies should be conducted to examine whether Baduanjin Qigong practice is able to produce improvements in leg power. Due to the limited number of studies, it is also not possible to infer which training parameters (frequency; duration) would more likely result in improvements in leg power.

As for trunk flexibility, the effects of Baduanjin Qigong on the sit-and-reach test were mostly favourable, with three of four studies [9, 34, 35] favouring Baduanjin Qigong over the control condition. The results thus indicate that Baduanjin Qigong practice can significantly enhance trunk flexibility. Based on the meta-analysis, at least five sessions/week with a minimum duration of 45 minutes of Baduanjin Qigong are recommended for improving trunk flexibility [35]. It is important to highlight that positive results on trunk flexibility were all observed in young adults [9, 34, 35]. Thus, we cannot confirm that similar results may be observed in older adults, which is usually a group that greatly benefits from good trunk flexibility, especially for performance of daily tasks. Future studies need to verify if the Baduanjin Qigong practice is also effective in improving flexibility of other regions of the body.

In this systematic review, we also included a meta-analysis on the effects of the Baduanjin Qigong practice on the six-minute walk test, which is commonly used to assess cardiopulmonary endurance in individuals with chronic respiratory disease and heart failure [61, 62]. The result indicates a favourable effect of the Baduanjin Qigong practice on the six-minute walk test. A possible explanation for the improvements in cardiopulmonary endurance is that the participants in the both two studies met a moderate level of intensity for an extended period during the regular Baduanjin Qigong routines [63]. For example, An et al. [2] reported five 30-minute Baduanjin Qigong sessions weekly for 8 weeks in 28 female patients with knee osteoarthritis and Xiao and Zhuang [41] reported four 45-minute Baduanjin sessions weekly for six months in 96 patients with mild-to-moderate Parkinson's disease. It is worth noting that, in both studies included in the meta-analyses, the participants presented conditions that are detrimental to locomotion, namely, knee osteoarthritis [2] and Parkinson's disease [41]. Therefore, the evidence of the true effects of Baduanjin Qigong on cardiopulmonary endurance is too scarce and requires further investigation.

### 4.4. Cardiovascular and Respiratory Parameters

Based on the results of nine studies, the effects of Baduanjin Qigong on systolic blood pressure and diastolic blood pressure were medium (SMD: −0.60; 95% CI: −0.94, −0.27) and small (SMD: −0.46; 95% CI: −0.73, −0.20), respectively. However, of the nine studies included, only three presented standardized mean differences that significantly favoured Baduanjin Qigong compared to the control condition when the outcome was systolic blood pressure [37–39]. Conversely, for diastolic blood pressure, only four of the nine studies significantly favoured Baduanjin Qigong compared to the control condition [17, 19, 37, 39]. It is worth noting though that for none of the studies the standardized mean difference significantly favoured the control condition compared to the Baduanjin Qigong practice. Therefore, it is likely that Baduanjin Qigong can attenuate both systolic and diastolic blood pressure independent of the age group, as favourable results were observed for young adults, adults, and older adults [17, 19, 37–39]. The results suggest that a minimum of three sessions of 30–60 minutes of practice is necessary for improving blood pressure [19]. Twelve weeks was the shortest intervention duration for observing benefits on systolic and/or diastolic blood pressure [38]. Patients with hypertension should follow regular drug treatment in conjunction with Baduanjin Qigong practice [38, 39]. Finally, the results of the present meta-analysis indicate that Baduanjin Qigong is effective in improving vital capacity and reducing resting heart rate. For vital capacity, the pooled standardized mean difference was significant and of medium magnitude (SMD: −0.77; 95% CI: −1.42, −0.11), favouring the effects of Baduanjin Qigong compared to the control condition. This result was based on six studies, of which only two demonstrated significant effects of Baduanjin Qigong on vital capacity [34, 35]. None of the other four studies favoured the control condition [9, 18, 20, 33]. The result is indicative that Baduanjin Qigong may be effective to improve vital capacity. In relation to resting heart rate, three of four studies presented significant standardized mean differences, resulting in a pooled standardized mean difference of −0.87 (95% CI: −1.47, −0.27), which denotes a large effect of Baduanjin Qigong on resting heart rate [16, 34, 35]. Thus, it is possible to affirm that Baduanjin Qigong is effective in lowering resting heart rate. This may be due to better autonomic control and body relaxation promoted by the systematic practice of Baduanjin Qigong. The studies showing positive results on resting heart rate only included college-age students. In these studies, participants practiced Baduanjin Qigong five times/week with durations varying from 45 to 60 minutes [35] and total intervention times ranging from 10 to 20 weeks [16, 34, 35]. Future studies should examine if Baduanjin Qigong also results in lower resting heart rate in older adults, which is a segment of the population that tends to already have lower values due to the aging process.

This study is not without limitations. Due to the limited number of studies investigating the effects of the Baduanjin Qigong on different health variables, we included studies with participants ranging from young adults to older adults and individuals with different health conditions. Thus, the lack of studies for specific age groups and health conditions limits our ability to more objectively determine the benefits of Baduanjin Qigong to health for each group. In addition, we only included studies published in English and Chinese languages, which certainly contributed to the limited number of studies included in this systematic review. One of the strengths of investigation is the quality control for study inclusion. We adopted the PEDRO scale in order to assess quality of the studies that were included in this systematic review.

## 5. Conclusion

The aggregated results from this systematic review show that Baduanjin Qigong practice is beneficial for quality of life, sleep quality, balance, handgrip strength, trunk flexibility, systolic and diastolic blood pressure, and resting heart rate. Further studies are necessary to confirm the effects of Baduanjin Qigong on leg power, cardiopulmonary endurance, and pulmonary function (e.g., vital capacity), while considering a long-term follow-up.

## Figures and Tables

**Table 1:** Summary of Baduanjin Qigong studies in the systematic review: randomized controlled trials.

Author, year	Study design	Sample size	Age, mean (SD) years	Study groups (pre/posttest)	Frequency and duration	Outcomes measured	Adverse events/follow-up
Li et al., 2015	RCT	222 healthy college students	20.78 (1.10)	BG (111/101)	BG: five one-hour Baduanjin Qigong sessions weekly for 12 weeks	Quality of life (WHOQOL); sleep quality (PSQI); physical health (step test; vital capacity; systolic and diastolic pressures; resting heart rate; sit-and-reach test for flexibility; standing long jump/m; handgrip strength)	NAE/12-week follow-up
				CG (111/105)	CG: keep original physical activity habit during the 12-week intervention period		

Wang, 2011	RCT	62 healthy college students	BG: 19.35 (0.88)	BG (27/27)	BG: three 60-minute Baduanjin Qigong sessions for 8 weeks	Quality of life (CSWQ)	Not mentioned/not mentioned
			CG: 19.20 (1.67)	CG (35/35)	CG: keep original lifestyle		

Y. Liu et al., 2012	RCT	88 patients with type 2 diabetes mellitus	BG: 62.64 (5.98)	BG (44/33)	BG: five 40-minute Baduanjin Qigong sessions weekly for 12 weeks	Quality of life (DMQLS)	Not mentioned/not mentioned
			CG: 65.64 (8.38)	CG (44/36)	CG: one 30-minute educational sessions relating to diabetes biweekly for 6 weeks		

Hu and Gu, 2014	RCT	109 healthy older adults with sedentary lifestyle	BG: 1 (6.87)	BG (57/55)	BG: four-to-seven 30–60-minute Baduanjin Qigong sessions for 6 months	Quality of life (SF-36); physical health (systolic and diastolic pressures; vital capability; handgrip strength; balance)	Not mentioned/not mentioned
			CG: 17 (8.04)	CG (54/54)	CG: keep original lifestyle		

Liu et al., 2014	RCT	108 patients with chronic diseases	BG: 67.1 (6.18)	BG (54/48)	BG: two 30-to-40-minute Baduanjin Qigong sessions weekly for 12 weeks, with a combination of home-based practice by watching Baduanjin Qigong DVD	Primary outcome: quality of life (SF-36)	Not mentioned/not mentioned
			CG: 66.63 (5.98)	CG (54/47)	CG: two 40-to-60-minute group walking weekly for 12 weeks, with a combination of self-walking at medium speed		

Xiu, 2015	RCT	70 patients with cancer	Not report	BG (36/36)	BG: regular care and Baduanjin Qigong exercise (two 30-minute sessions per day for 8 weeks, with seven days weekly)	Quality of life (EORTC-QLQ-C30)	Not mentioned/not mentioned
				CG (34/34)	CG: regular care		

Li et al., 2014	RCT	40 patients with type 2 diabetes	BG: 53.6 (8.7)	BG (20/20)	BG: seven 30-minute Baduanjin Qigong sessions weekly for 4 weeks	Sleep quality (PSQI)	Not mentioned/not mentioned
			CG: 51.4 (9.2)	CG (20/20)	CG: educational sessions *relating to diabetes mellitu*s		

Chan et al., 2014	RCT	150 patients with chronic fatigue syndrome-like illness	BG: 19.1 (7.8)	BG (75/65)	BG: 16 90-minute Baduanjin Qigong sessions for nice consecutive weeks	Sleep quality (PSQI)	No adverse events/three-month follow-up
			CG: 38.9 (8.1)	CG (75/65)	CG: keep original lifestyle		

Wang et al., 2009	RCT	60 patients with type 2 diabetes	Mean age of 57.8	BG (30/23)	BG: not mentioned the frequency but 2 months	Sleep quality (PSQI)	Not mentioned/not mentioned
				CG (30/29)	CG: keep original lifestyle. All three groups had regular healthcare		

Xiao and Zhuang, 2016	RCT	96 patients with mild-to moderate Parkinson's disease	67.53 (8.56)	BG (48/45)	BG: four 45-minute sessions weekly and daily walking 30 minutes for six months	Sleep quality (Parkinson's Disease Sleep Scale 2 (PDSS2)); balance (BBS); mobility (TUG); 6-minute walking	Not mentioned/not mentioned
				CG (48/44)	CG: daily walking for 20 minutes		

Chen et al., 2012	RCT	60 patients with essential hypertension	BG: age ranges from 62 to 73	BG (30/30)	BG: educational lessons and Baduanjin Qigong training (three-to-four Baduanjin Qigong sessions weekly, with 1-hour training twice per day)	Sleep quality (PSQI); blood pressure (systolic and diastolic pressures)	Not mentioned/not mentioned
			CG: age ranges from 60 to 75	CG (30/30)	CG: hypertension-related educational lessons		

Li et al., 2014	RCT	110 healthy sedentary adults	BG: 35.5 (16)	BG (55/46)	BG: more than three 30–60-minute Baduanjin Qigong sessions weekly for 16 weeks	Physical health: sit-and-reach test for flexibility; stork balance test; aerobic endurance; blood pressures (systolic and diastolic pressures); vital capacity	Not mentioned/not mentioned
			CG: 32.9 (13)	CG (55/55)	CG: keep original lifestyle		

Zheng et al., 2014	RCT	60 old patients with essential hypertension (EH) grade one	BG: 69.23 (3.72)	BG (30/27)	BG: five 30-minute Baduanjin Qigong sessions weekly for 12 weeks; regular drug treatment and care	Diastolic and systolic pressures	Not mentioned/not mentioned
			CG: 70.06 (3.51)	CG (30/28)	CG: regular drug treatment and care		

Qiu et al., 2014	RCT	100 healthy college students	Age ranges from 18 to 25	BG (50/50)	BG: five 50-minute Baduanjin Qigong sessions weekly for 18 weeks	Handgrip strength; sit-and-reach test for flexibility; stork balance; standing long jump; aerobic endurance (step test); blood pressures (systolic and diastolic pressures); vital capacity; resting heart rate	Not mentioned/not mentioned
				CG (50/50)	CG: keep original lifestyle		

Zhai et al., 2013	RCT	44 healthy older adults with sedentary lifestyle	BG: 64.9 (2.5)	BG (22/22)	BG: five 40-to-50-minute Baduanjin Qigong sessions weekly for eight weeks	Handgrip strength; stork balance test; vital capacity; blood pressures (systolic and diastolic pressures)	Not mentioned/not mentioned
			CG: 64.8 (2.7)	CG (22/22)	CG: keep original lifestyle		

An et al., 2008	RCT	28 female patients with knee osteoarthritis	BG: 65.4 (8.2)	BG (14/11)	BG: five 30-minute Baduanjin Qigong sessions (taped command) weekly for 8 weeks	6-minute walk test	Not mentioned
			CG: 64.6 (6.7)	CG (14/10)	CG: regular healthcare		

A. L. Liu et al., 2012	RCT	60 college students with a score of PSQI ≥ 8	Not mentioned	BG (30/30)	BG: five 45-to-60-minute Baduanjin Qigong sessions weekly for 10 weeks	Handgrip strength; stock balance test; sit-and-reach test; vital capacity; resting heart rate	Not mentioned/not mentioned
				CG (30/30)	CG: keep original lifestyle		

Pan and Feng, 2010	RCT	48 patients with essential hypertension (EH) grade one	BG: 62.1 (5.8)	BG (24/24)	BG: ten 45-minute Baduanjin Qigong sessions weekly for 24 weeks (twice per day), plus regular drug treatment	Systolic and diastolic pressures	Not mentioned/not mentioned
			CG: 61.4 (7.1)	CG (24/24)	CG: regular drug treatment		

Qin, 2012	RCT	20 healthy college students	Age ranges from 21 to 22	BG (10/10)	BG: five 60-minute Baduanjin Qigong sessions weekly for 20 weeks	Resting heart rate; systolic and diastolic pressures	Not mentioned/not mentioned
				CG (10/10)	CG: keep original lifestyle		

(1) Baduanjin Qigong group = BG; CG = control group.

(2) RCT = randomized controlled trial.

(3) WHOQOL = World Health Organization Quality of Life Scale; SF-36 = Short Form (36) Health Survey; DMQLS = quality of life scale for patients with type 2 diabetes mellitus; CSWQ = College Students Well-being Questionnaire; EORTC-QLQ-C30 = The European Organization for Research and Treatment of Cancer Quality of Life Questionnaire-C30; PSQI = Pittsburgh Sleep Quality Index; BBS = Berg Balance Test.

(4) NAE = no adverse events; NM = not mentioned about adverse events or follow-up intervention.

**Table 2:** PEDro scales of included randomized *controlled* trials.

Study	Eligibility criteria	Random allocation	Concealed allocation	Similar at baseline	Subject blinded	Therapists blinded	Assessor blinded	<15% dropout	Intention-to-treat analysis	Between-group comparisons	Points measures and variability	Total
Li et al., 2015	1	1	1	1	0	0	1	1	1	1	1	8/10
Wang, 2011	1	1	0	1	0	0	1	1	1	1	1	7/10
Y. Liu et al., 2012	1	1	0	1	0	0	1	0	0	1	1	5/10
Hu and Gu, 2014	1	1	0	1	0	0	0	1	0	1	1	5/10
Liu et al., 2014	1	1	0	1	0	0	1	1	0	1	1	6/10
Xiu, 2015	1	1	0	1	0	0	1	1	1	1	1	7/10
Li et al., 2014	1	1	0	1	0	0	1	1	1	1	1	7/10
Chan et al., 2014	1	1	0	1	0	0	1	1	1	1	1	7/10
Wang et al., 2009	1	1	0	1	0	0	1	0	0	1	1	5/10
Xiao and Zhuang, 2016	1	1	0	1	0	0	1	1	1	1	1	7/10
Chen et al., 2012	1	1	0	1	0	0	1	1	1	1	1	7/10
Li et al., 2014	1	1	0	1	0	0	1	0	0	1	1	5/10
Zheng et al., 2014	1	1	0	1	0	0	0	1	0	1	1	5/10
Qiu et al., 2014	1	1	0	1	0	0	0	1	1	1	1	6/10
Zhai et al., 2013	1	1	0	1	0	0	0	1	1	1	1	6/10
An et al., 2008	1	1	0	1	0	0	0	0	0	1	1	4/10
A. L. Liu et al., 2012	1	1	0	1	0	0	1	1	1	1	1	7/10
Pan and Feng, 2010	1	1	0	1	0	0	0	1	1	1	1	6/10
Qin, 2012	1	1	0	1	0	0	0	1	1	1	1	6/10

0 = does not meet the criteria; 1 = meet the criteria. Criteria (without eligibility criteria) were used to calculate the total PEDro score.
